# Directed movement toward, translocation along, penetration into and exit from vascular networks by breast cancer cells in 3D

**DOI:** 10.1080/19336918.2021.1957527

**Published:** 2021-08-02

**Authors:** Deborah J. Wessels, Claude Pujol, Nikash Pradhan, Daniel F. Lusche, Luis Gonzalez, Sydney E. Kelly, Elizabeth M. Martin, Edward R. Voss, Yang-Nim Park, Michael Dailey, Sonia L. Sugg, Sneha Phadke, Amani Bashir, David R. Soll

**Affiliations:** a Developmental Studies Hybridoma Bank and W.M. Keck Dynamic Image Analysis Facility, Department of Biology, The University of Iowa, Iowa City, IA, USA; b Department of Surgery, The University of Iowa Hospitals and Clinics, Iowa City, IA, USA; c Department of Internal Medicine, The University of Iowa Hospitals and Clinics, Iowa City, IA, USA; d Department of Pathology, The University of Iowa Hospitals and Clinics, Iowa City, IA, USA

**Keywords:** Filopodia, pseudopod, intravasation, extravasation, computer-assisted reconstruction, CD44, RHAMM

## Abstract

We developed a computer-assisted platform using laser scanning confocal microscopy to 3D reconstruct in real-time interactions between metastatic breast cancer cells and human umbilical vein endothelial cells (HUVECs). We demonstrate that MB-231 cancer cells migrate toward HUVEC networks, facilitated by filopodia, migrate along the network surfaces, penetrate into and migrate within the HUVEC networks, exit and continue migrating along network surfaces. The system is highly amenable to 3D reconstruction and computational analyses, and assessments of the effects of potential anti-metastasis monoclonal antibodies and other drugs. We demonstrate that an anti-RHAMM antibody blocks filopodium formation and all of the behaviors that we found take place between MB-231 cells and HUVEC networks.

## Introduction

Metastatic breast cancer is a leading cause of cancer mortality in women [1–3]. The major mechanism of metastasis in all cancers [4], including breast cancer [5], is thought to occur via intravasation into the peripheral blood [6], and/or the lymphatics [7], by tumor cells that have undergone the epithelial to mesenchymal transition [8]. Upon intravasation, the circulating tumor cells [9] disseminate to distant sites [10], where they extravasate to seed secondary tumors [11,12]. Intravasation of cancer cells into vessels has been visualized by intravital imaging [13] and identified in histological preparations of breast cancer [14]. Extravasation has also been visualized in *in vitro* models in which endothelial tubules are formed in microfluidic chambers [15]. *In vivo*, intravasation has been shown to require contact between an endothelial cell, a macrophage and a cancer cell [14], creating a doorway, referred to as the ‘Tumor MicroEnvironment of Metastasis’ [6,16], through which the cancer cell intravasates. Reports, primarily from observations of fixed tissue from melanoma cases [17,18], but also from prostate cancer [19] and cutaneous squamous cell carcinoma [20], suggest that cancer cells can also crawl along the outside walls of blood capillaries [17,21]. The interactions between cancer cells and endothelial cells, therefore, have received intense scrutiny at the molecular level [22–24], but less so at the behavioral level, which has the potential to identify new molecular targets that can be pursued to block metastasis.

To this end, we have begun to develop a computer-assisted 4D tumorigenesis model to facilitate the study of cell interactions related to tumorigenesis and metastasis [25,26]. Using this model, we previously observed by computer-assisted 3D reconstruction that cells from a variety of tumorigenic cancer cell lines, as well as cancer cells from fresh tumor tissues, actively aggregate and coalesce when seeded in a 3D Matrigel matrix, a behavior not observed in preparations of nontumorigenic cells [26–28]. These cancer cell-specific behaviors were mediated by filopodia and pseudopodia, and involved specialized physical interactions [26–28]. We then expanded this model to investigate in 3D the behavioral interactions of fibroblasts and cancer cells, and found that fibroblasts, activated by cancer cell-conditioned media, accelerated coalescence of cancer cells through reciprocal signaling and direct cell interactions [29]. Fibroblast networks also acted as scaffolds for cancer cell aggregation.

Here, we have used this computer-assisted 3D model to investigate the behavioral interactions of tumorigenic, breast cancer-derived MDA-MB-231 (MB-231) cells and human umbilical vein endothelial cells (HUVECs). A 2D HUVEC reticulated multicellular network, supported by a Matrigel cushion was overlayed with a 3D Matrigel matrix containing MB-231 cells evenly dispersed in 3D. Using optical sectioning and 3D-reconstruction of live cells over time, we observed that MB-231 cells migrated in a directed fashion onto the static HUVEC scaffold. Directional migration was mediated by filopodia extending from MB-231 cells suggesting a tactile mechanism of tracking. The filopodia that connected to HUVECs expanded to accommodate the cell body cytoplasm as the latter tracked the path of the filopodia to the contacted HUVEC. MB-231 cells, when fully attached to the HUVEC networks, crawled in a persistent fashion along the branches of the network, forming transient aggregates. At times, the MB-231 cells penetrated the HUVEC networks, migrated within the multicellular HUVEC networks, and then exited the HUVEC networks and continued migrating on the HUVEC network surfaces. Adhesion of cancer cells to the HUVECs did not block pseudopod-driven cell migration, even when contact to HUVEC cells was over the entire 3D surface of the cancer cells. The basic model can now be expanded to include combinations of breast cancer cells, fibroblasts, endothelial cells, macrophages, and the formation of HUVEC tubes [15]. The transparent 3D model allows for computer-assisted 3D reconstruction at time intervals of cell interactions associated with tumorigenesis and metastasis, and is highly amenable to assessing the potential blocking activity of antibodies, as shown here for antibodies against CD44 and the receptor of hyaluronate mediated motility [26,28–32].

## Methods

### Growth and maintenance of cell lines and primary cells

Human umbilical vein endothelial cells (HUVECs) and basal endothelial cell growth medium (EBM-2), supplemented with the Endothelial SingleQuots Kit, were obtained from Lonza (Basel, Switzerland). EBM-2 and the SingleQuots Kit were mixed to make the complete endothelium growth medium, EGM-2. MB-231 breast cancer cells were obtained from ATCC and cultured for 12–15 passages in MCF medium, which consisted of DMEM/F12 basal medium (Life Technologies, Carlsbad, CA) supplemented with 5% horse serum, human recombinant EGF, insulin, hydrocortisone and cholera toxin, all obtained from Sigma Aldrich (St. Louis, MO), and penicillin-streptomycin from Thermo-Fisher (Grand Island, NY) [33]. GFP-tagged MB-231cells, obtained from Angio-Proteomie (Boston, MA), were cultured according to the supplier’s directions. MCF-10A, a non-tumorigenic cell line derived from normal breast epithelial cells [34], was purchased from the American Type Culture Collection (Manassas, VA) and cultured in mammary epithelial cell basal medium, as described elsewhere [26].

### Generation of MDA-MB-231/EGFP for injection into mice

To generate MDA-MB-231/EGFP, MB-231 cells were transfected with the plasmid pEGFP-C3 (BDBiosciences, San Jose, CA). The plasmid was linearized at the AseI restriction site and used to transfect cells using FuGENE HD Transfection Reagent (Promega, Madison, WI; https://www.promega.com/) according to the supplier’s specifications. Stable clonal transfectants were obtained by selection with 330 µg/ml of G418 disulfate (Sigma, St. Louis, MO).

### Preparation of HUVEC networks

A 20 mm glass insert in the dish bottom of a 35 mm plastic Petri dish (Cellvis, Mountain View, CA), was coated with 200 µl of Matrigel and placed in a 37°C, 5% CO_2_ incubator for 30 minutes to allow Matrigel gelation [26]. A suspension of 5 × 10^5^ HUVECs in 200 µl of EGM-2 was then plated on the Matrigel layer. After 4 hours of incubation at 37°C in 5% CO_2_, the HUVECs had formed a characteristic reticulated network [35]. 2D images at 4x or 10x magnification were acquired through an Olympus CK2 microscope housed in an incubator at 37°C and 5% CO_2_ as previously described [29].

### Preparation of 3D samples

**Figure 1.**
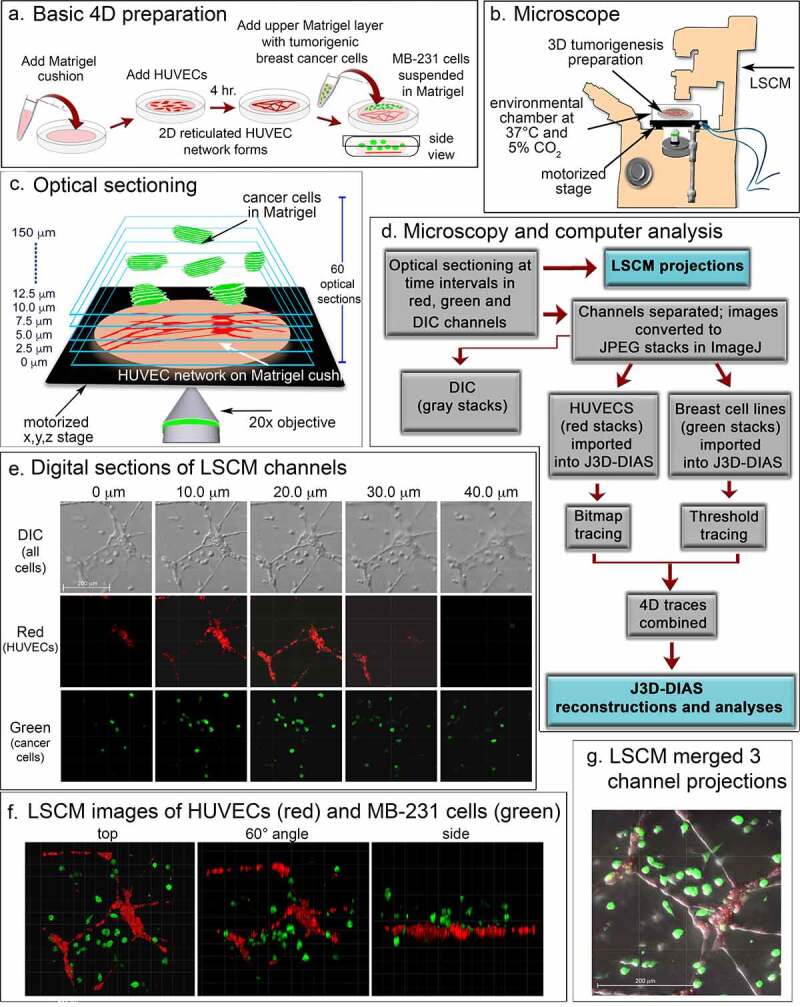
A transparent 3D preparation was employed to assess the behavior of live breast cancer cells in the vicinity of a reticulated network of endothelial cells. (a) A schematic diagram of the basic preparation. A suspension of human umbilical vein endothelial cells (HUVECs) plated on a matrigel cushion formed a 2D reticulated multicellular network on the cushion surface after 4 hours at 37°C. The network was then overlayed with a suspension of MB-231 cells suspended in matrigel at 5°C. After one hour at 37°C, the Matrigel polymerized, resulting in a deep matrigel overlay containing randomly dispersed MB-231 cells. (b) A set of optical sections of deepRed dyed HUVECs and GFP-MB-231 cells were acquired at time intervals for up to 72 hours through a 20X objective using HyD detectors at 638 and 488 nm, respectively, along with DIC images. Preparations were housed in an environmental chamber at 37°C in 5% CO_2_ on the stage of a Leica TCS SP8 laser scanning confocal microscope (LSCM) with a motorized x,y,z stage. (c) Sixty optical sections were acquired through 150 μm at 2.5 μm intervals. (d) Flowchart of microscopy and computer-assisted reconstruction. (e) Representative z-series from the DIC, red and green channels for an early preparation obtained from the Leica TCS SP8 at 10 μm intervals. (f) LSCM Z-projections from each channel were merged for preliminary analysis at three angles. (g) Three channels were merged to generate a single image projection viewed from on top

To vitally stain HUVECs red, a stock solution of Cell Tracker DeepRed dye (Invitrogen, Waltham, MA) was prepared by dissolving 15 µg in 50 µl of DMSO and the stock solution stored at −20°C. HUVECs were harvested at 70–80% confluency and 5 × 10^5^ cells resuspended in 1 ml of RPMI medium without serum. One µl of the Deep Red stock solution was added and the cell suspension incubated for 30 minutes at 37°C in 5% CO_2_. The dyed HUVECs were then pelleted, resuspended in 200 µl of EGM-2, plated on the Matrigel-coated glass insert of a 35 mm Petri dish and incubated for 4 hours. Excess medium and unattached HUVECs were carefully removed by gently washing the insert with EGM-2. The periphery of the insert was dried with a sterile cotton swab. MB-231-GFP cells were harvested at 70–80% confluency. A suspension of 5 × 10^5^ cells in 100 µl of MCF medium was mixed with 500 µl of ice-cold Matrigel as previously described in detail [26]. A 200 µl aliquot was then carefully pipetted over the HUVEC network and incubated for one hour at 37°C in 5% CO_2_ to polymerize the Matrigel (Figure 1(a)). MCF medium was then added to the dish. For non-tumorigenic MCF-10A cells, cells from a culture at 70–80% confluency were suspended in RPMI medium containing 22 µM CellTracker^TM^ orange CMRA Dye (Invitrogen, Waltham, MA) at a concentration of 5 × 10^5^ cells/mL. The mixture was incubated at 37°C in 5% CO_2_ for 30 min. Cells were then pelleted at 100 g for 4 min, resuspended in 100 µl of MCF medium, mixed with Matrigel and plated over the HUVECs as described above. To test the effects of select monoclonal antibodies on MB-231 cell behavior, 20 μl of a mAb was added to the cell suspension, or medium without cells, to obtain a final volume of 100 µl, then mixed with 200 µl of Matrigel. This mixture was distributed over the HUVEC cell network on the Matrigel cushion. For studies on the effects of antibodies, the 300 µl mixtures contained 150 µg of the anti-CD44 mAb H4C4 (Developmental Studies Hybridoma Bank [DSHB], https://dshb.biology.uiowa.edu/), and/or 20 µg of the anti-HMMR/RHAMM polyclonal antibody 35,000,002 (Novus Biologics, Centennial, CO). The H4C4 mAb was purified following a previously described protocol [31]. Briefly, H4C4 was purified from hybridoma supernatant by using Protein G HP SpinTrap columns (GE Healthcare, Chicago, IL) and concentrated in DPBS (Gibco, Waltham, MA) using Amicon ultra-centrifugal filter units (EMD MilliporeSigma, Burlington, MA).

### 4D image acquisition

Images were acquired using the Leica TCS SP8 confocal microscope located in the Roy J. Carver Center for Imaging in the Department of Biology at the University of Iowa. The dish containing the preparation was placed in an environmental chamber that maintained the sample at 37°C in 5% CO_2_, throughout image acquisition (Figure 1(b)). Z-series images were acquired for 7–9 fields per sample, employing the precision motorized xyz stage at 2.5 um steps, every 10 minutes for up to 72 hours through a 20x objective (Figure 1(c)) using 488, 552 and 638 nm laser lines for green, yellow and red fluorescent markers, respectively. Images in the Leica file format from each channel (DIC, red, green and yellow) were converted to JPEG images using ImageJ [36] (Figure 1(d,e)). Direct reconstructions of the red and green channels were performed using the Leica Application Suite (LAS X) software (Figure 1(f)). JPEG images from each channel were also converted into z-projections which could be merged using ImageJ to generate a movie that could be viewed in QuickTime (Figure 1(g)).

**Figure 2.**
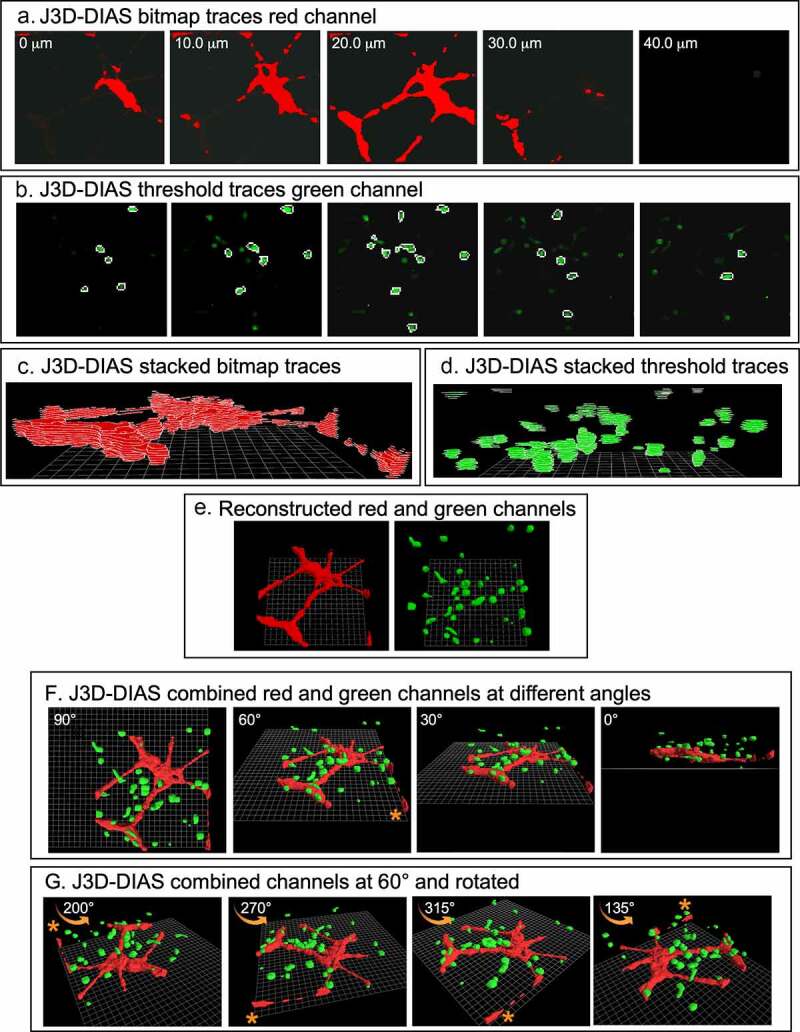
3D reconstructions using J3D-DIAS4.2 computer program. (a) Bitmap segmentation of HUVECs. (b) Threshold segmentation of GFP-231 cells. (c) Stacked bitmap series of HUVEC networks. (d) Stacked threshold series of cells. (e) J3D-DIAS reconstructed red (HUVEC) and green (MB-231) channels. (f) Combined red (HUVEC) and green (MB-231) J3D-DIAS reconstructions viewed at different angles. (g) Rotation of combined red and green channels reconstructed at a 60° angle. The asterisk in f and g is a reference point for rotation in panel G

J3D-DIAS4.2 [25,29,32] was used for accurate and precise quantitative analysis of cell movements and shapes. To reduce the size of files and increase the speed of analysis, JPEG images were imported into J3D-DIAS4.2 [25] as described above (Figure 1(d)) and saved as movies in the DIAS format. Image segmentation of HUVEC networks was performed automatically using a bitmap algorithm (Figure 2(a)) that retains gaps and spaces in the network [26]. MB-231 and MCF-10A cells were automatically outlined using a threshold detection algorithm [37–39] (Figure 2(b)). Overlapping bitmap and edge traces of the optical sections were stacked in the z-axis (Figure 2(c,d)), respectively). Bitmap pixels were expanded into voxels and the voxel blocks wrapped into a continuous surface [26], while the pixel-based outlines of objects detected by thresholding were replaced with beta-spline models [25]. J3D-DIAS4.2 then built a faceted surface from the beta-splines using the ‘marching cubes’ algorithm [40]. Path files in both cases were constructed from the position of the 3D centroid at 10 minute intervals [25] and velocity data calculated from the centroid track [32,41]. The surface area of HUVEC networks was calculated from the faceted reconstructions [25]. HUVEC surface area was determined for nodes with contiguous branches and percent decrease calculated relative to the 4 hour network. A node was defined as an area with at least 3 branches. Nodes with branches that had detached from the network were excluded. Reconstructed images from the red and green channels were viewed separately in J3D-DIAS4.2 (Figure 2(e)) or combined and viewed from different angles and rotations (Figure 2(f,g)). The proportions of MB-231-GFP cells contacting HUVECs were counted manually using these reconstructions.

### Generation of MB-231 CD44°^e^

MB-231 cells were transformed with the plasmid pLX304 expressing CD44 isoform 12 under the CMV promoter and a C-terminal V5 tag as previously described [33,42]. In brief, 5 × 10^4^ cells were plated in each well of a 12 well plate and allowed to attach overnight in growth medium. The medium was then replaced with OptiMEM (Life Sciences, Carlsbad, CA) for 24 h. To transform cells, the medium was changed to 500 µl OptiMEM medium supplemented with 20 µl of medium containing 5 µl of Fugene (Promega, Madison, WI), 2 µg of pLX-304-CD44 isoform 12 and 98 µl of OptiMEM. Cells were allowed to grow overnight and maintained in 10 µg/ml selection drug Blasticidin S (Enzo Life Sciences, NY).

### Immunostaining

Cells were immunostained as described elsewhere in detail [31]. Briefly, cells were grown overnight on a coverslip in a 20 mm Petri dish in growth medium, rinsed the following day and fixed in 4% paraformaldehyde (Electron Microscopy Sciences, Hatfield, PA). Preparations were blocked in 1% BSA in TBS, pH7.6, prior to treatment with 10 μg/mL of the anti-CD44 mAb H4C4 (DSHB, http://dshb.biology.uiowa.edu and/or the anti-RHAMM rabbit polyclonal. Following rinses, preparations were counterstained with Alexa Fluor 488 goat anti-mouse IgG (Jackson ImmunoResearch, West Grove, PA) to visualize CD44 and goat-anti rabbit Alexa 647 (Jackson ImmunoResearch, West Grove, PA) to visualize RHAMM.

### Western blot analysis

Western blot analysis was performed as previously described [43]. To detect RHAMM expression, rabbit anti-RHAMM (ThermoFisher Scientific) was employed as primary antibody. Mouse anti-hGAPDH (DSHB; http://dshb.biology.uiowa.edu) antibody was used for loading control detection. IRDye 800-conjugated goat anti-rabbit or goat anti-mouse antibody (Li-Cor Biosciences, Lincoln, NE) was used as a secondary antibody. Odyssey scanner and software were used for detection and quantification of immunoblots (Li-Cor Biosciences). RHAMM expression levels were normalized to the GAPDH loading control and values were determined from three separate experiments.

### Acquisition, culturing and vital dye staining of fresh human tissue

Fresh human breast cancer tissue and matched normal control tissue were obtained through The Breast Molecular Epidemiology Resource (BMER) through the University of Iowa BMER study (IRB 20,100,379), an Institutional Review Board-approved biospecimen repository, and through approved Informed Consent protocols signed by patients who chose to enroll. Tissue samples were dissected into fragments and placed in the wells of a 6 well plate in MCF medium. Once cell growth was apparent, medium was exchanged every 3 days. Cells were harvested from the wells at 70–80% confluency by trypsinization and transferred into T-75 tissue culture flasks. A stock solution of CellTracker™ Green CMRA (Thermo Fisher Scientific, Waltham, MA) was prepared by dissolving 50 µg of the powder in 40 µl DMSO, and the solution was stored at −20°C. The cells were harvested at 70–80% confluency, and 5 × 10^5^ cells were centrifuged at 800 g for 10 min. The cell pellet was resuspended in 1000 µl of RPMI medium without serum, followed by the addition of 1.0 µl of the stock solution of Green CMRA dye. The cell suspension was incubated for 30 minutes at 37°C in 5% CO_2_. The dyed cells were then pelleted and resuspended in MCF medium and ice cold Matrigel as described above. After gentle mixing, the suspension was plated atop a HUVEC network, the latter prepared as described above, and incubated for 60 minutes at 37°C in 5% CO_2_. MCF medium was then added to the dish.

### Mouse injections, harvesting tumors, cryosectioning and cryostaining

All studies using mice were approved by the University of Iowa Institutional Animal Care and Use Committee (IACUC) under Protocol 8,121,508 and in compliance with Public Health Service Policy, Animal Welfare Regulations and the Guide for the Care and Use of Laboratory Animals. Female NOD.CB17-PRKDCSCID/J mice were obtained from Jackson Labs and injected at 6–8 weeks of age in the mammary fat pad with 2 × 10^6^ MB-231-GFPcells suspended in 200 µl of sterile PBS. Tumors that developed from GFP-MB-231 cells were harvested and fixed in 4% paraformaldehyde for formalin-fixed paraffin embedded (FFPE) sectioning or embedded in optimal cutting temperature (OCT) compound for cyrosectioning. FFPE sections were H&E stained. To identify endothelial cells, OCT sections were stained with a 1:20 dilution of anti-PECAM (CD31) monoclonal antibody P2B1 (Developmental Studies Hybridoma Bank (DSHB) http://dshb.biology.uiowa.edu). Images were acquired in the University of Iowa Central Microscopy Research Facilities utilizing a Zeiss 710 confocal microscope.

## Results

### Experimental strategy

**Figure 3.**
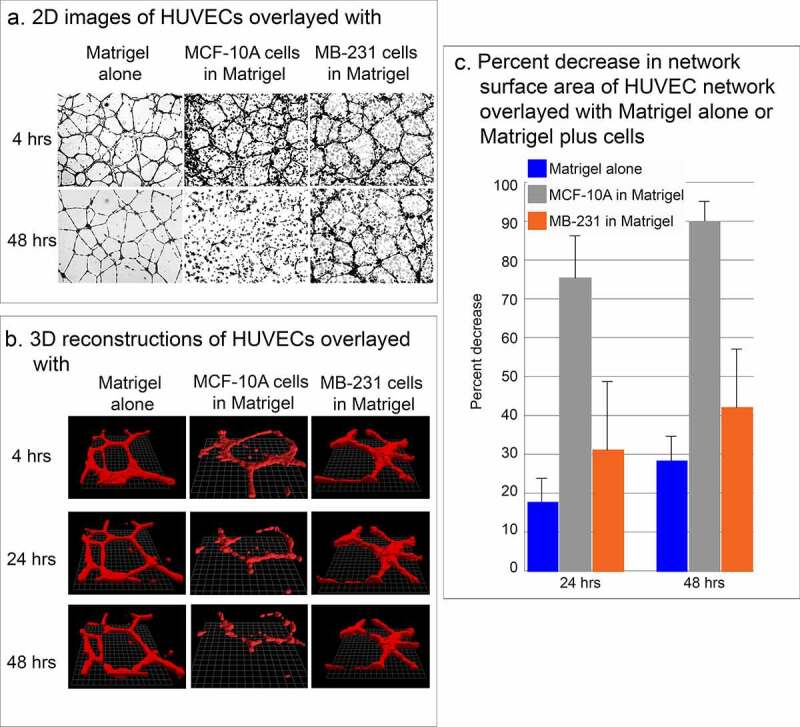
Stability of the HUVEC network in the presence of MB-231 and MCF-10A cells. (a) Low magnification 2D brightfield images of the HUVEC network viewed at 4 and 48 h, overlayed with Matrigel alone, Matrigel containing cells of the nontumorigenic breast epithelial cell line MCF-10A, or Matrigel containing MB-231 cells. (b) J3D-DIAS reconstructions of HUVEC networks at 4, 24 and 48 hrs, alone, overlaid with MCF-10A cells or overlaid with MB-231 cells. (c) Percent decrease of network surface area in the HUVEC network was determined using J3D-DIAS bitmap reconstructions as described in the Methods

The basic preparation for analyzing in 3D the behavior of tumorigenic MB-231 cells in the vicinity of a HUVEC network is diagrammed in Figure 1(a). To generate this preparation, first a transparent thin lower Matrigel cushion was cast in a culture dish, and a HUVEC suspension then dispersed on top of the cushion. By 4 h, a 2D reticulated network of HUVECs formed atop the Matrigel cushion (Figure 1(a)). The nodes and branches were multicellular, and the branches appeared as multicellular chords several cells thick. A suspension of MB-231 cancer cells in cold, unpolymerized Matrigel, was then pipetted over the HUVEC network, and the temperature increased to 37°C, causing rapid polymerization of the Matrigel upper layer, approximately 150 µm thick containing the randomly dispersed MB-231 cells. The preparation was then positioned on the motorized stage of a laser scanning confocal microscope (Figure 1(b)) and optically sectioned at 2.5 μm increments, obtaining 60 optical sections through 150 μm in a two-minute period (Figure 1(c)). The sections were imaged through three channels, a differential interference contrast (DIC) channel, which visualized both HUVECs and MB-231 cells, a red channel which visualized exclusively HUVECs which were stained with DeepRed dye, and a green channel which visualized exclusively GFP-expressing MB-231 breast cancer cells (Figure 1(d)). Optical sectioning was repeated every 10 minutes, providing a 4D (3D movie) presentation for analysis. Multiple regions of the preparation were optically sectioned at each time point. In Figure 1(e), an example of optical sections at one time point, at 10 μm increments through 40 μm, is presented for one region of the preparation. In Figure 1(f), Figure 3(d) LSCM reconstructions were viewed at three angles. In Figure 1(g), a top view example is presented of an image produced by combining z-projections of the DIC, green and red LSCM channels. To generate computer-assisted 3D reconstructions, the red and green channels were imported into J3D-DIAS and saved in the DIAS format [25,26,32,41,44]. Optical sections of the HUVEC network (red channel) were traced by bitmap algorithms (i.e., raster graphics) [45] (Figure 2(a,c)), while optical sections of the cancer cells (green channel) were traced by threshold algorithms [45] (Figure 2(b,d))). The optical sections in each case were then connected in the z-axis using J3D-DIAS software [32,41,44,46] (Figure 2(e)). The two reconstructions (red HUVEC network and green cancer cells) were then combined at each time point, and could be viewed at different angles (Figure 2(f)) and/or rotated (Figure 2(g)) to assess visually or quantitatively behavioral interactions in 3D.

### Stability of HUVEC network

The effectiveness of assessing over time the physical interactions of MB-231 cells in the vicinity of the HUVEC network hinged on the stability of the network. When HUVEC networks were overlaid with Matrigel devoid of cells, the networks remained relatively stable for at least two days (48 hours) (Figure 3(a,b)). When HUVEC networks were overlaid with Matrigel containing cells of the nontumorigenic, breast derived, noncancer cell line MCF-10A [34], the network disassembled (Figure 3(a,b)). When HUVEC networks were overlaid with Matrigel containing cells of the tumorigenic breast cancer cell line MB-231, the HUVEC network was relatively stable for at least 48 hours, exhibiting some dissociation, but far less than that exhibited under Matrigel containing MCF-10A cells (Figure 3(a,b)). These observations were supported by computer-assisted measurements of the surface areas of networks converted to mathematical models by bitmap tracing at 24 and 48 hours of incubation. In the absence of cells in the upper Matrigel layer, the surface area of the multicellular HUVEC network decreased on average by 17% and 28% by 24 and 48 hours, respectively (Figure 3(c)). In the presence of nontumorigenic MCF-10A cells in the upper Matrigel layer, however, the surface area of the multicellular HUVEC network decreased on average by 75% and 90% by 24 and 48 hours, respectively (Figure 3(c)). In the presence of MB-231 cells, the decrease in surface area was 31% and 42% by 24 and 48 hours, respectively (Figure 3(c)), which, as we show, was sufficient for analyses of network interactions with MB-231 cells.

### MB-231 cells move toward and attach to HUVEC networks

**Figure 4.**
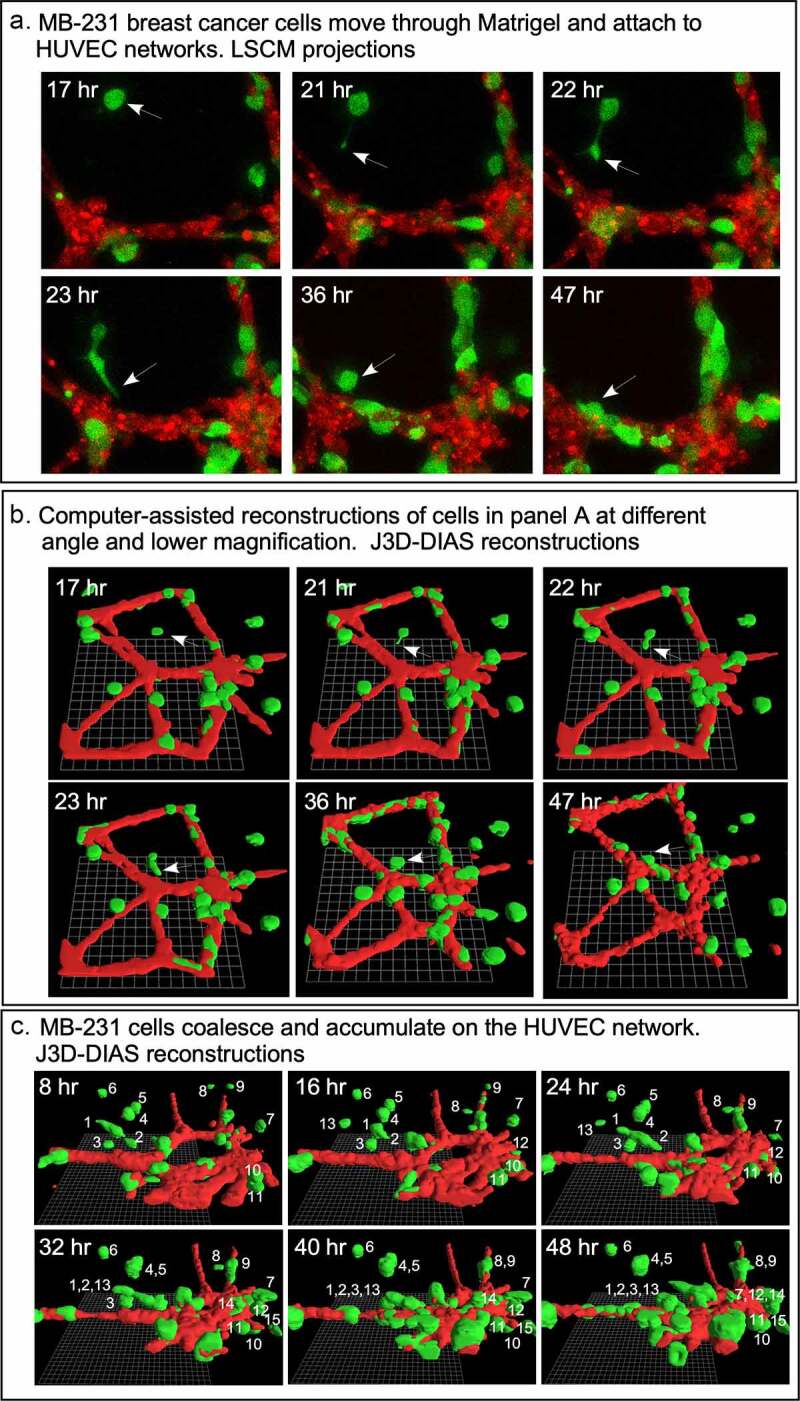
MB-231 cells (green) in proximity of a HUVEC network (red) accumulate over time on the network. (a) LSCM projections viewed from on top. (b) J3D-DIAS reconstruction viewed from on top. Arrow points to a cell moving onto a HUVEC network. (c) J3D-DIAS reconstruction from a side angle. In panel C, cells are numbered to follow their trajectory over time

**Figure 5.**
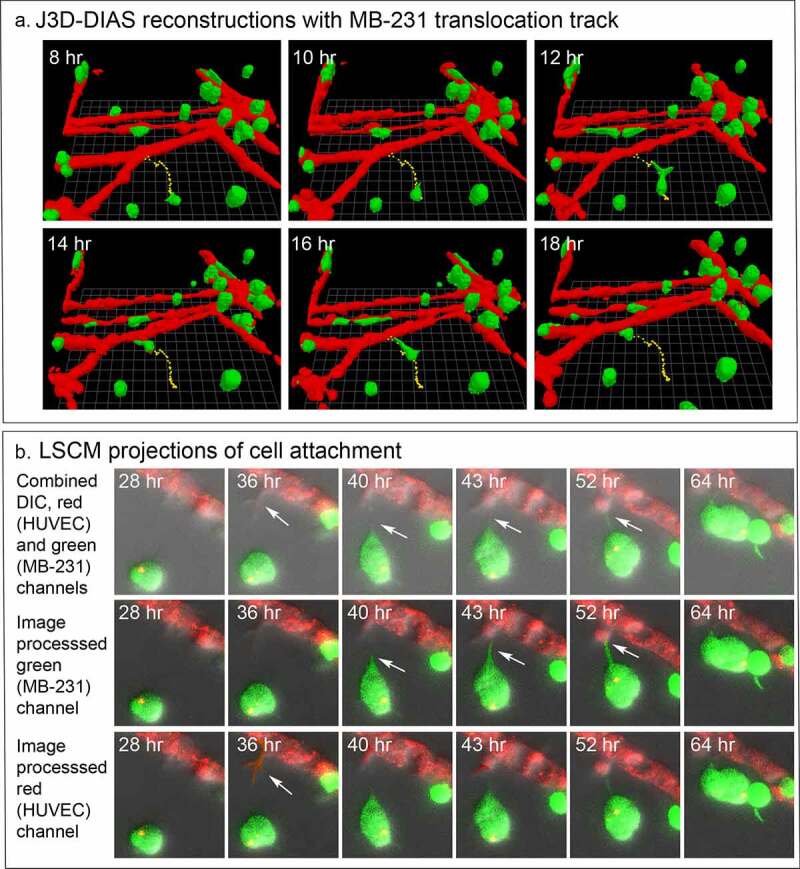
The majority of MB-231 cells moved in a persistent fashion toward the HUVEC network, forming filopodia that expanded into pseudopods in the direction of the network. HUVEC cells targeted by the MB-231 cells, in turn extended a projection at the site of contact with the MB-231 projection. (a) The translocation track of a MB-231 cell moving in the direction of a HUVEC network. The cell body is positioned along the track as a function of time. The network and cell are J3D-DIAS reconstructions. (b) The formation of an anterior filopodium by an MB-231 cell contacting a cell in the HUVEC network and the protrusion (pointed to by white arrow) from the HUVEC cell in response. The network and cell are LSCM projections. Red, HUVEC network; green, MB-231 cell; yellow, centroid track

**Figure 6.**
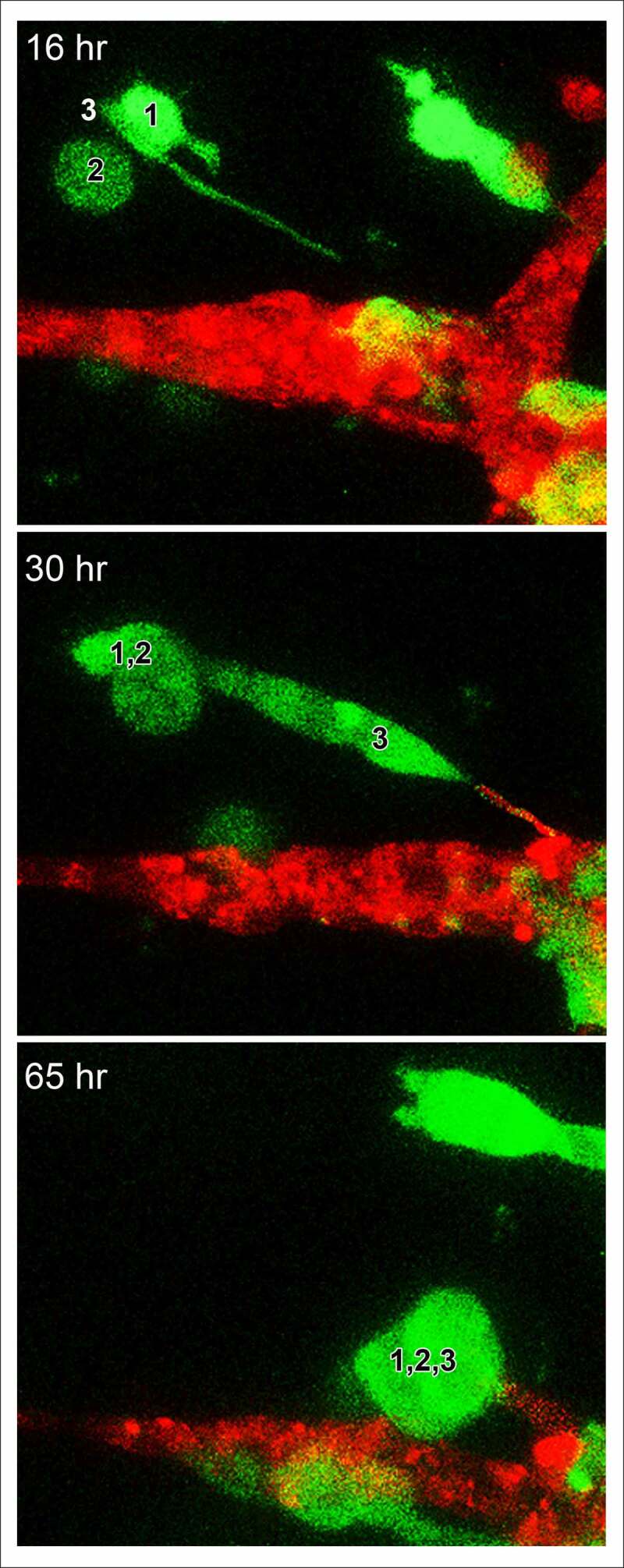
A second, higher magnification example of an LSCM image of a MB-231 filopodium directed toward a HUVEC network, cell aggregation (cells 1,2,3) prior to contact with the network, and a protrusion by the targeted HUVEC cell in response to the MB-231 filopod. Time sequence between 16 and 65 hours of incubation. Green, MB-231 cell; red, HUVEC network

For each preparation of MB-231 cells (green) and a HUVEC network (red), we generated LSCM Z-projections, as in Figure 4(a), and, using J3D-DIAS software, generated computer reconstructions, as in Figure 4(b,c)). The regions of the preparation analyzed included the HUVEC network on the Matrigel cushion and the 3D region of the thick overlaid Matrigel region, containing dispersed MB-231 cells in proximity to the network. The depth of the reconstructed region was approximately 150 μm. The diameter of the average MB-231 cell body was 30 µm. The LSCM 3D images facilitated assessments of cellular details, such as lamellipodia, pseudopodia, filopodia and the shape of the cell body, while J3D-DIAS computer reconstructions facilitated a clearer view of spatial relationships and measurements of changes in cellular morphology and translocation in time. Several characteristics of the behavior of MB-231 in proximity to HUVEC networks were evident (see Supplemental movie). By 47 to 48 hours of incubation, the majority of MB-231 cells in the proximity of the HUVEC network had moved actively and directionally toward the HUVEC network, extending pseudopodia in the direction of the network (Figure 4(a,b))). In Figure 4(a,b)), the same MB-231 cell, noted by a white arrow, extended a pseudopod toward the network, then the cell body followed the trajectory toward the network. By 47 to 48 hours, the majority of cells originally in the proximity of the HUVEC network had migrated to and adhered to the HUVEC network (Figure 4(a-c)). Many cells from outside the imaged region moved into the region and also accumulated on the HUVEC network (Figure 4(a-c)). In Figure 4(c), numbered cells can be tracked over a 40 hour period onto a HUVEC network. In Figure 5(a), a representative example is presented of the biased trajectory of the centroid (center of mass) of an MB-231 cell in the direction of the HUVEC network. In this example, the entire track is presented at each time point, but the position of the cell body (green) is presented along the track at the time indicated on each panel. The average velocity of MB-231 cells translocating toward HUVEC networks was 0.40 μm/min (standard deviation 0.08 μm/min, N = 50). The majority of MB-231 cancer cells initially in the proximity of the HUVEC network moved to and adhered to the network by 36 hours (Figure 4(a-c)). 68% of MB-231 cells (N = 100) within 65 μm of the network translocated toward and adhered to the network, whereas 35% of MB-231 cells (N = 100) at distances ≥ 80 μm, did not translocate or translocated in random directions. The difference was significant (p-value 0.006 Student T-test). Close scrutiny of LSCM images revealed that MB-231 cells approximately two or more cell diameters from the HUVEC network and translocating toward the HUVEC network appeared to be connected to the networks by a single filopodium, just at the threshold of LSCM resolution. Filopodia have been shown to be as thin as 0.2 µm, or less, and as long as 70 µm or more [47], increasing the difficulty of reconstruction. In Figure 5(b), an example is presented of a MB-231 two cell aggregate attached to a HUVEC network by a filopodium, that tracked the filopodium as the aggregate translocated toward the network. The MB-231 filopodium contracted as the MB-231 aggregate approached the HUVEC network, as is accentuated in the time series of images processed for the green (MB-231) channel (Figure 5(b)). At the filopodium contact point on the targeted HUVEC, a projection was extended by the HUVEC, which was accentuated in the 36 through 52 hour images processed for the red (HUVEC) channel (Figure 5(b)). In Figure 6, a second example is presented of a MB-231 three cell aggregate tracking a filopodium onto a HUVEC network. At 16 hours, the aggregate extended a filopodium toward the network. By 30 hours, the cytoplasm of cell 1 had translocated into the filopodium which was in contact with a projection extended from the target HUVEC (Figure 6). By 65 hours, the elongated cells had contracted, drawing the MB-231 cell aggregate to the surface of the HUVEC network (Figure 6). These results demonstrate a complex set of cell interactions facilitated by a filopod of the MB-231 cell and a projection of the HUVEC cell, that are involved in the directed translocation of the MB-231 cell to the HUVEC network.

### Cells of the nontumorigenic breast cell line MCF-10A do not accumulate on HUVEC networks

**Figure 7.**
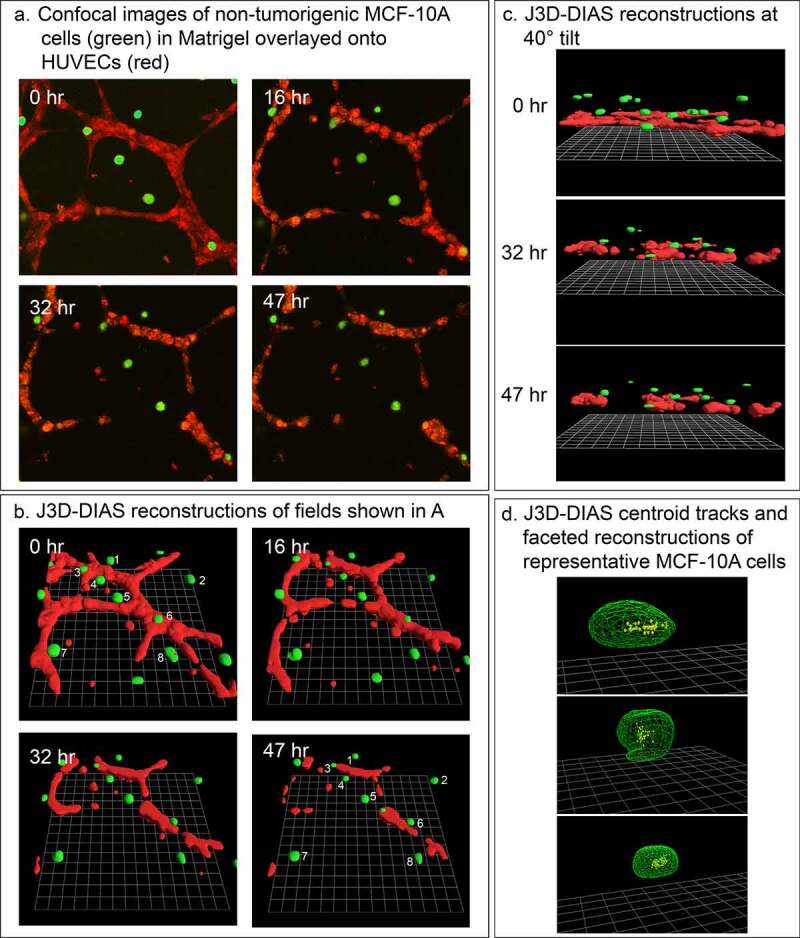
Breast-derived nontumorigenic MCF-10A cells do not translocate in a directed fashion toward HUVEC networks, supporting the contention that tumorigenic MB-231 cells attach to HUVEC networks in an active and directed movement, not by random collisions. MCF-10A cells not only fail to move to the HUVEC network, but also signal dissociation of the HUVEC network by a soluble factor. (a) View from on top of an LSCM projection of MCF-10A cells and a HUVEC network over a 47 hour period of incubation. (b) View at an angle of J3D-DIAS reconstructions of the preparation in panel A. Cells translocating along the HUVEC network are numbered. (c) View from the side of J3D-DIAS reconstructions of the preparation in panel A. (d) An example of centroid tracks of representative MCF-10A cells in the proximity of the HUVEC networks. MCF-10A cells are color-coded green in panels A–C

We previously demonstrated that nontumorigenic breast-derived cell lines and cells from fresh normal breast tissue do not translocate significantly or form aggregates in Matrigel models of tumorigenesis [26–28]. To support the suggestion that the behavior of MB-231 cells in the proximity of HUVEC networks may be specific to tumorigenic cells and involves directed movement rather than random collisions, we analyzed the behavior of cells of the nontumorigenic mammary-derived non-cancer cell line MCF-10A in the proximity of HUVEC networks. In our preparations (Figure 1(a)), the MCF-10A cells remained nonmotile and did not accumulate on the HUVEC networks (Figure 7(a-c)). The lack of motility is evident in a comparison of the positions of cells 1 through 8 at 0 and 47 hours, respectively, in the preparation in Figure 7(b). Centroid tracks of MCF-10A cells were clustered over the 47-hour period of analysis, suggesting random changes in direction due to shape changes, not active, persistent translocation (Figure 7(d)). It should be noted that the HUVEC network disaggregated over the 47 hours of analysis (Figure 7(a-c)), as noted in Figure 3(b). Similar results were obtained with MCF-7 cells [48], a nonaggressive breast cancer cell line with low metastatic potential [49]. These results support the suggestion that the accumulation of MB-231 cells on HUVEC networks involves directional, active migration that may be specific to aggressive tumorigenic breast cells.

### Behavior of MB-231 cells attached to HUVEC networks

**Figure 8.**
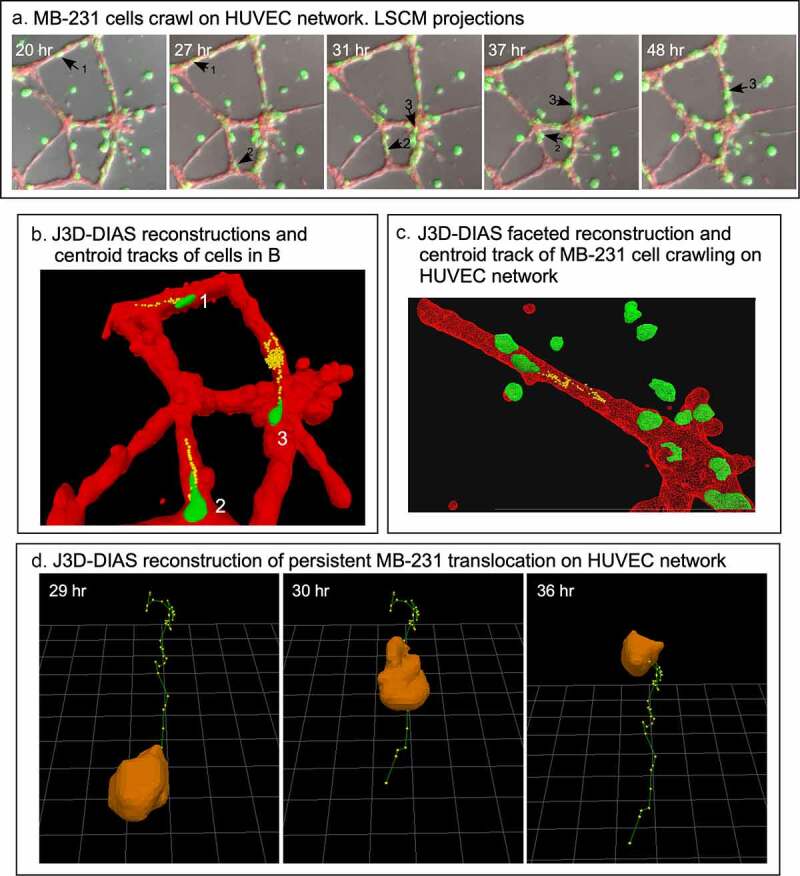
MB-231 cells attached to HUVEC networks translocate along the network branches in a persistent, directed fashion. (a) Merged LSCM projection images of MB-231 over time, with select translocating cells numbered. (b) J3D-DIAS generated centroid tracks plotted at 10 minute intervals of MB-231 cells translocating along HUVEC branches (cell 1 and 2) and node (cell 3) of a HUVEC network. (c) A second example of a J3D-DIAS generated centroid track of an MB-231 cell translocating along a HUVEC branch. The cell and HUVEC branch were reconstructed using facets. (d) Centroid track of a solitary MB-231 cell plotted at 10 minute intervals. The cell reconstruction is presented at the beginning of the track in panels B and C and at time points in d

In Figure 8(a), LSCM Z-projections of numbered cells over a 28 hour period of analysis reveal the extent of translocation of three numbered cells [1–3]. MB-231 cells not only translocated to HUVEC networks, but they also adhered to and translocated along the branches and nodes of the network. Once an MB-231 cell adhered to a HUVEC, it rarely released, suggesting strong adhesive interactions. Of 100 cancer cells that migrated to and then adhered to a HUVEC network, 0% detached in the subsequent 17 to 36 hours of incubation. Translocation by the adherent cells was sporadic, switching between persistent, directed translocation along the branches, and stationary periods of no net translocation (Figure 8(a,b))). The stationary periods were accompanied by changes in cell shape that led to small, random changes in the position of the clustered cell centroids (Figure 8(b,c)). For the centroid tracks in Figure 8(b,c)), the cell body was reconstructed at the beginning of each track. The rate of persistent single cell translocation averaged 0.73 μm/min (± 0.31 N = 20), twice the velocity of MB-231 cells moving in a directed fashion toward a HUVEC network in Matrigel. In most cases, the translocating cell was tapered at its anterior end (Figure 8(b,c)). In Figure 8(d)), we windowed in the absence of the supporting HUVEC network, the track of a single MB-231 cell translocating on the surface of the HUVEC network, to accentuate the cone-shaped anterior end of the translocating cell along the persistent portion of the track. In many cases, attached MB-231 cells clustered along the HUVEC network (Figure 4(a)), but cells exited these clusters, suggesting weaker attachments between MB-231 cells contacting HUVECs than between cells of tumorigenic cell lines aggregating in Matrigel in the absence of other cell types [26–29]. Increased clustering of MB-231 cells at nodes compared to branches may simply be the result of the increase in area of the adhesive substrate.

### MB-231 cells transiently penetrate into, migrate within, exit from, and continue migrating on HUVEC networks

**Figure 9.**
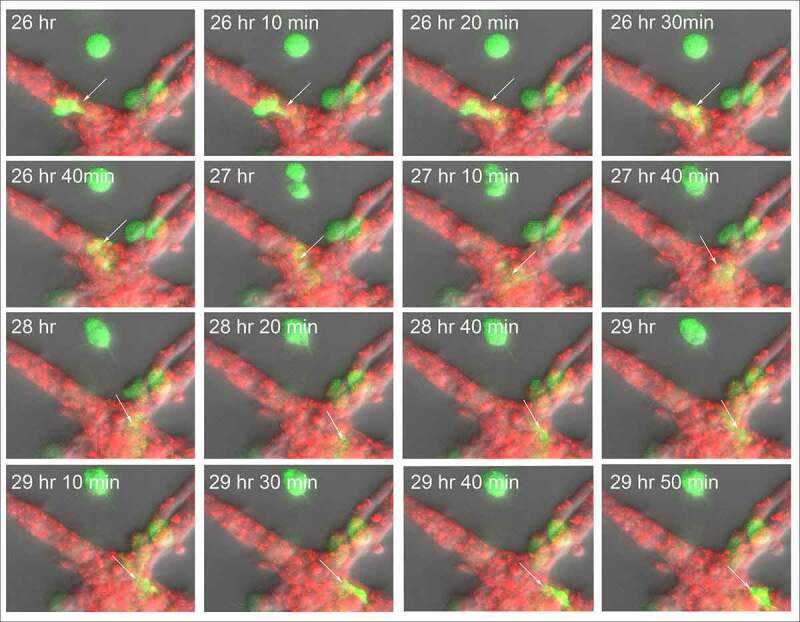
An example of an MB-231 cell that penetrates a HUVEC branch, crawls through the inside of a node, exits the node at an opposing branch and finally resumes migration along the HUVEC branch. Penetration is mediated by an anterior pseudopod, as is exits. A white arrow points to the cell of interest. LSCM projection images are presented

**Figure 10.**
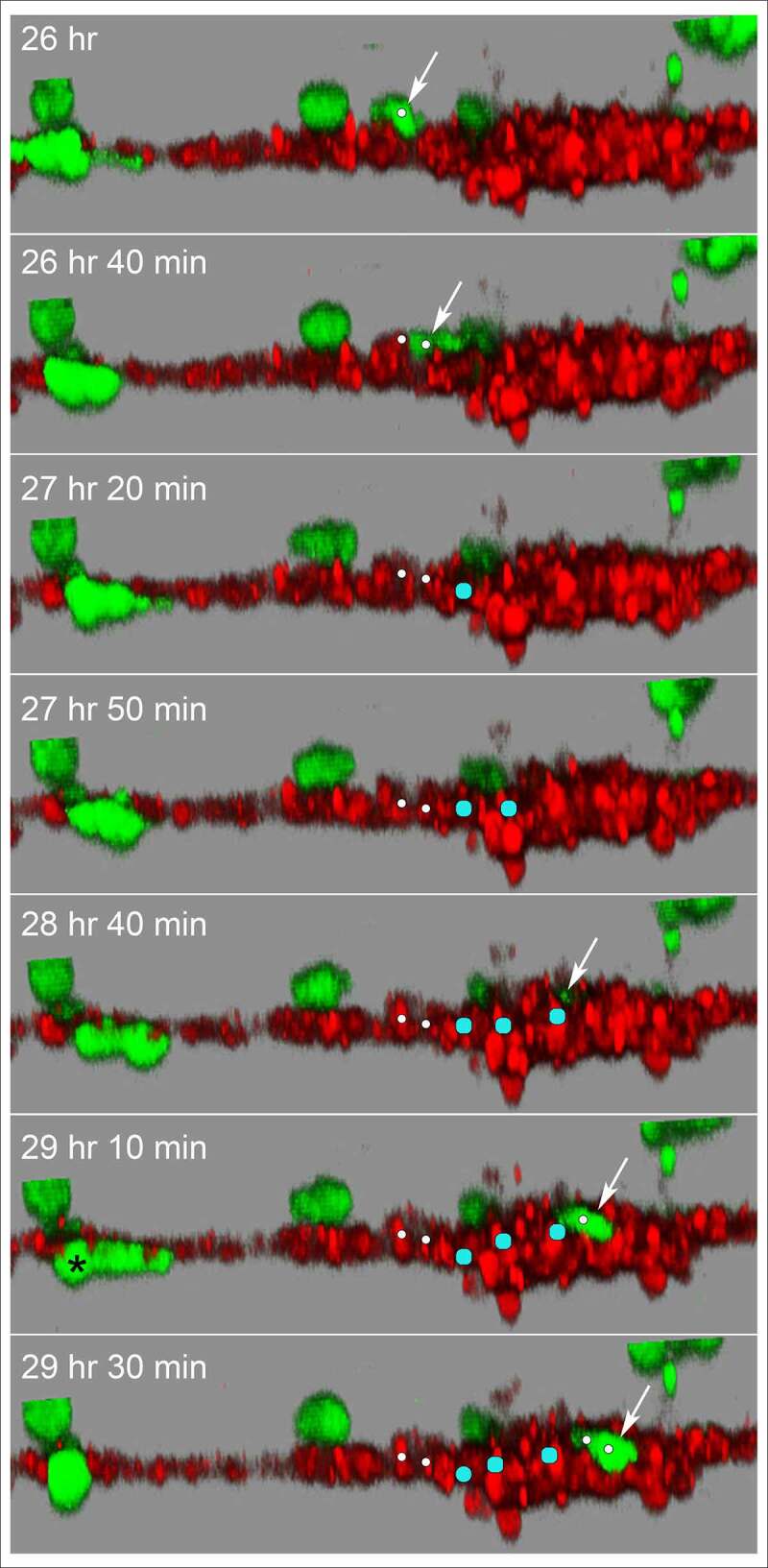
Side view of an MB-231 cell translocating along, penetrating into, translocating inside, exiting and translocating again along a HUVEC network. White arrow points to the position of the MB-231 cell when on the surface of the HUVEC network. Blue dot is the inferred position of the MB-231 cell when the majority of the cell body was inside the HUVEC cell mass. The LSCM images were selected from a sequence generated over three and a half hours. MB-231 cell labeled with an asterisk (*) at 29 h 10 min moves under the HUVEC branch

Although the HUVECs in our model form a multicellular reticulated network of nodes and branches devoid of tubes, we found numerous examples of MB-231 cells penetrating the HUVEC cables and nodes, crawling through the stacked HUVECs, and then reemerging, as shown in the LSCM Z-projection images combining the red (HUVEC) and green (MB-231) channels in Figure 9. The penetrating green MB-231 cell, pointed to by a white arrow, extended an anterior pseudopod over a 30 minute period (26 hr to 26 hr 30 min), which penetrated the HUVEC node (Figure 9(a)). The cell body followed the pseudopod into the HUVEC node, becoming less distinct and yellow due to the red HUVEC cells overlying the green MB-231 cell (26 hr 30 min to 28 hr; Figure 9). The MB-231 cell migrated through the HUVEC node for 1 hour and 20 minutes (26 hr 40 min to 28 hr), then extended a pseudopod out of the HUVEC node at 28 hr 20 min and fully migrated out onto the surface of the HUVEC node (28 hr 20 min to 29 hr 10 min; Figure 9). The MB-231 cell continued migrating atop the HUVEC network (29 hr 10 min to 29 hr 50 min). The cell migrated approximately five cell diameters in three hours and 50 minutes (Figure 9). In Figure 10, we have tracked a MB-231 cell migrating into, through and out of a HUVEC node, imaged by side views of LSCM 3D images. The centroid track is presented as small white dots when the cell was on the surface of the HUVEC network and by large blue dots when migrating within the HUVEC node (Figure 10). Again, the MB-231 cell penetrated the HUVEC layer, migrated through the layer, bounded on all sides by HUVEC cells, then exited to the surface of the HUVEC layer (Figure 10). The rate of translocation was actually faster within the HUVEC layer than in Matrigel. In the same preparation, a cell marked with a black star, moved under the HUVEC layer (Figure 10).

### Antibodies against CD44 and RHAMM

**Figure 11.**
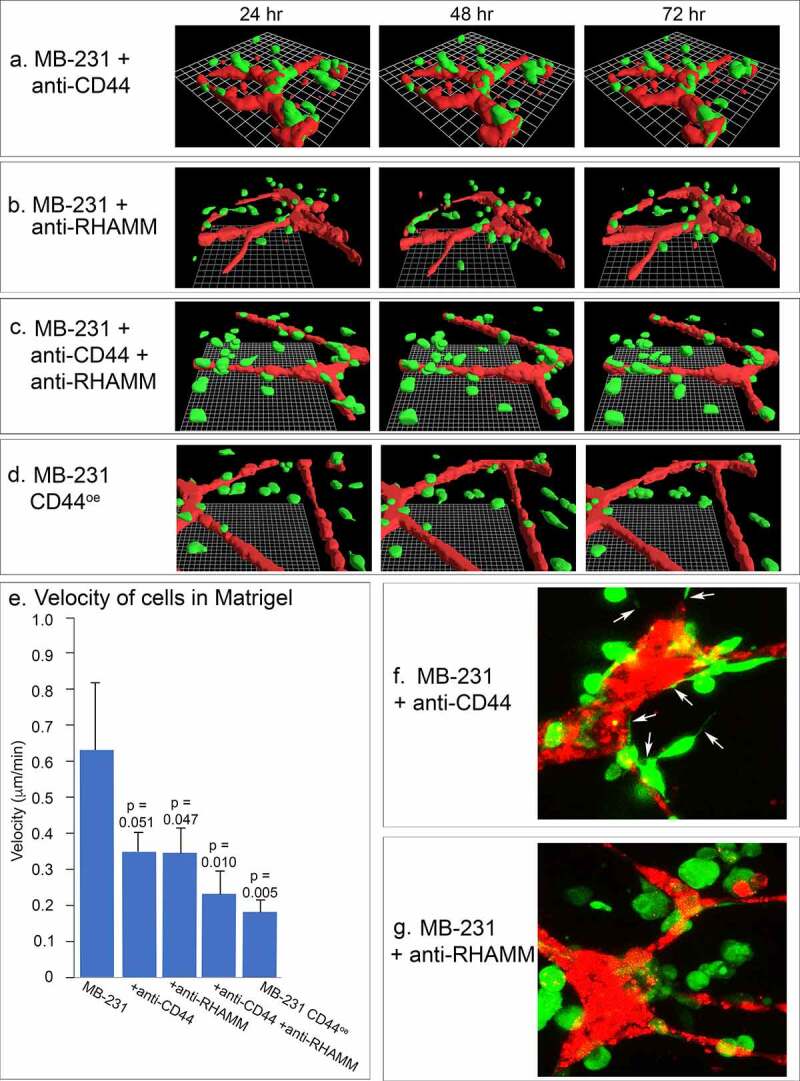
The effects of anti-CD44 and anti-RHAMM on the behavior of MB-231 cells in the proximity of a HUVEC network. (a,b,c) J3D-DIAS reconstructions of MB-231 cells and HUVEC networks, between 24 and 72 hours of incubation, treated with anti-CD44 mAb (H4C4), anti-RHAMM pAb (anti-RHAMM/CD168), and a combination of the two Abs, respectively. (d) J3D-DIAS reconstructions of MB-231 cells overexpressing CD44 (MB-231 CD44°^e^) in proximity of HUVEC networks. (e) Velocity of MB-231 cells, antibody treated cells and CD44 overexpressor cells in the vicinity of a HUVEC network. Single cell velocity of untreated MB-231 cells, and MB-231 cells treated with anti-CD44 mAb, anti-RHAMM pAb, a combination of the two antibodies, and MB-231 cells overexpressing CD44. Values provided are means ± standard deviations. *P*-values against normal, untreated MB-231 cells were computed by Student’s t-test. (f) Representative MB-231 cells treated with anti-CD44 mAb form filopodia toward HUVECs. (g) Representative MB-231 cells treated with anti-RHAMM pAb do not form filopodia toward HUVECs

The behavioral studies of MCF-10A cells in the proximity of HUVEC networks suggested that accumulation on the networks by MB-231 cells was due to active and directed migration, rather than random collisions. To explore this point further, we tested the effects of Abs against two cell surface molecules that play central and interactive roles in cancer cell migration and adhesion, CD44 [27,46,47] and RHAMM [30,33,34]. Neither the anti-CD44 mAb H4C4, the anti-RHAMM polyclonal antibody anti-RHAMM/CD168, or a combination of H4C4 and anti-RHAMM/CD168, affected the stability of the HUVEC networks over a 48 hour period, between 24 and 72 hours of incubation (Figure 11(a-c)), respectively), to a degree that would affect the analysis of the behavior of MB-231 cells in the proximity of HUVEC networks. Anti-CD44 did not block the migration toward and adhesion to the HUVEC network by MB-231 cells as is evident in the 48 hour reconstruction period after migration and adhesion in Figure 11(a), but anti-RHAMM alone or in combination with the anti-CD44 mAb blocked both migration toward and adhesion to the HUVEC network (Figure 11(b,c)), respectively). Either antibody, or a combination of the two, did cause a decrease in velocity of cells translocating in Matrigel (Figure 11(d)). The p-values, using the Student’s T-test, for the comparison of anti-CD44, anti-RHAMM and anti-CD44/anti-RHAMM treated preparations with untreated preparations were 0.051, 0.047 and 0.010, suggesting that decreases in velocity in Matrigel were significant. More noteworthy was the effect on filopodium formation. Whereas the anti-CD44 mAb had no effect on the extension of filopodia from MB-231 cells to the HUVEC network (Figure 11(f)), the anti-RHAMM mAb completely blocked filopod extension (Figure 11(g)). These latter results support the suggestion that filopodia play a major role in the directed movement of MB-231 cells toward HUVEC networks, and this process is dependent on the cell surface molecule RHAMM. Quantitative analysis of western blots performed in triplicate revealed a 60% increase in RHAMM expression in MB-231 cells as compared to the level in MCF-10A cells, which behave like MB-231 cells treated with anti-RHAMM antibodies, supporting the suggestion that RHAMM plays a role in orchestrating MB-231 interactions with the HUVEC network.

In addition to inhibition studies with anti-CD44 and anti-RHAMM Abs, we analyzed the behavior of a transformant of MB-231 that overexpressed CD44 isoform12, MB-231 CD44°^e^. Western blots probed with the anti-CD44 mAb, H4C4, and immunostaining revealed that CD44°^e^ cells expressed approximately twice as much CD44 as parental MB-231 cells (supplementary Figure S1A). In parental MB-231 cells, both CD44 and RHAMM localized to the plasma membrane (supplementary Figure S1B). In MB-231 CD44°^e^ cells, however, while CD44 localized to the plasma membrane, RHAMM abnormally relocalized in the cytoplasm, leaving the MB-231 cell surface devoid of RHAMM (supplementary Figure S1B). MB-231 CD44°^e^ cells did not migrate or adhere to HUVEC networks (Figure 11(d) and supplementary Figure S1C), and exhibited a 70% decrease in velocity (Figure 11(e)). They also did not extend filopodia. These results support the suggestion that cell surface RHAMM is essential for directed movement and adherence to a HUVEC network by MB-231 cells.

### Fresh breast tumor cell behavior

**Figure 12.**
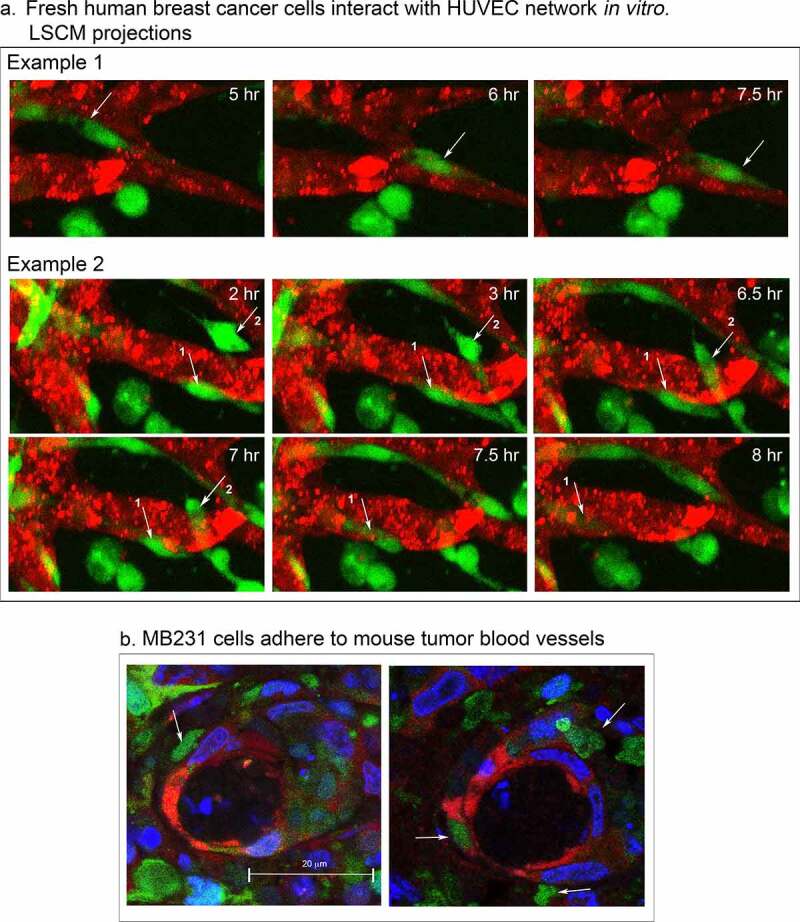
Fresh human breast cancer cells interact with HUVEC networks like MB-231 cells, and MB-231 cells in an MB-231 tumor generated in a mouse interact with tumor blood capillaries. (a) Fresh human breast cancer cells vitally stained with Green CMRA (green) in the proximity of a HUVEC network (red). Example 1, a human cancer cell translocating on the surface of a HUVEC branch. Example 2, human breast cancer cells penetrating the network (arrows point to the two cells). (b) Histological sections of MB-231 cells adhering to blood vessels in a tumor formed by MB-231 cells in a mouse mammary fat pad. MB-231 cells express GFP constitutively (green); nuclei are stained blue with DAPI; blood vessel cells are stained red with an anti-PECAM mAb

To assess whether the complex behaviors of MB-231 cells interacting with HUVEC networks were specific to the test cell line, or a general feature of breast cancer cells, we analyzed cancer cells from fresh human mammary tumors from three separate patients in our experimental preparations. It should be noted that both CD44 [50,51] and RHAMM [52] are expressed on the cell surface of primary breast cancer cells. Tumor cells from all three patient samples migrated through Matrigel to the HUVEC networks, adhered to the HUVEC cells, migrated along the surfaces of the network, and penetrated the networks, in a fashion similar to MB-231 cells. In Figure 12(a), Example 1, a breast cancer cell, noted by a white arrow, migrated on the surface of the HUVEC network one cell length in 2.5 hours, and in Figure 12(b), Example 2, two breast cancer cells noted by white arrows penetrated HUVEC network branches. These results indicate that the behavioral interactions of MB-231 cells with HUVEC networks are representative of the behavior of fresh breast cancer cells.

### *MB-231 cells interact with tumor blood capillaries* in vivo

To test whether MB-231 cells interact with blood capillaries in tumors formed *in vivo*, we analyzed tumors formed in mammary fat pads of mice by MB-231 cells. The MB-231 cells were transfected with a GFP-expressing plasmid. When palpable tumors formed after four weeks, mice were euthanized, the tumors excised, fixed, cryosectioned and stained with both the anti-PECAM mAb CD31, P2B1, to visualize endothelial cells (red) [53] and DAPI to visualize nuclei (blue). Transfected MB-231 cells fluoresced green. Although green MB-231 cells were distributed throughout the tumor, they also attached to the outer surface of blood vessels in the mouse mammary fat pad tumor (Figure 12(b)). The MB-231 cells bound to capillaries were morphologically asymmetric, a characteristic of motile MB-231 cells attached to HUVECs in our *in vitro* preparations.

## Discussion

Interactions between cancer cells and endothelial cells play two major roles in cancer progression. First, they play a role in the vascularization of tumors, in order to supply nutrients and oxygen, and remove metabolic waste and CO_2_, processes necessary for tumor viability and growth [54–56]. Second, interactions between cancer and endothelial cells play a major role in metastasis [57]. Metastatic cells shed from tumors migrate to and intravasate blood vessels, a process facilitated by macrophages [58]. Intravasated cancer cells are then swept by blood flow to specific anatomical locations, where they extravasate through the vessel walls, entering body locations to form secondary tumors [5,59]. While there has been intense investigation of the regulatory, cell surface and signal molecules involved in adhesion during both the vascularization of tumors [60–64] and cancer cell intravasation of blood and lymph vessels [65–70], the dynamic behavior of interacting cancer and endothelial cells in a 3D matrix has received much less attention. This deficit is in part due to the underutilization of computer-assisted 3D reconstruction systems of live cells over time. We have, therefore, begun to develop transparent 3D preparations, and LSCM and computer-assisted reconstruction techniques, that facilitate *in vitro* investigations of cellular behaviors basic to tumorigenesis and metastasis. We first applied these methods to analyze cancer cells in a transparent 3D Matrigel environment in the absence of other cell types, and identified specialized behaviors and unique cell types which mediated aggregate coalescence preceding spherule formation [26,28]. We also demonstrated using this model that a minority of cancer cells can recruit nontumorigenic cells into the tumor-like aggregates, a possible explanation for the cellular heterogeneity of tumors [27]. The model was then used to test the activity of 266 mAbs primarily against cell surface molecules, for their ability to block aggregation in a 3D Matrigel matrix [31]. Of the 266 tested mAbs, only those against the two components of integrin α3β1 and against CD44 exhibited blocking activity [31]. Recently, the model was employed to assess interactions between breast cancer cells and fibroblasts [29]. The results revealed both reciprocal signaling and direct physical interactions [29]. Here, we have used this general model to assess the behavioral interactions between breast cancer cells and multicellular reticulated networks of human umbilical vein endothelial cells (HUVECs). In contrast to our previous studies, we added laser scanning confocal microscopy, which allowed us to import optical sections of differentially colored MB-231 cells and HUVECs through multiple channels. These images could then be used to generate LSCM 3D images of live cells over time, or by bitmap algorithms and edge detection, 3D reconstructions of the HUVEC network and MB-231 cells over time. The latter J3D-DIAS reconstructions could be used to quantitate behavior.

### MB-231 cell behavior in the 3D model

**Figure 13.**
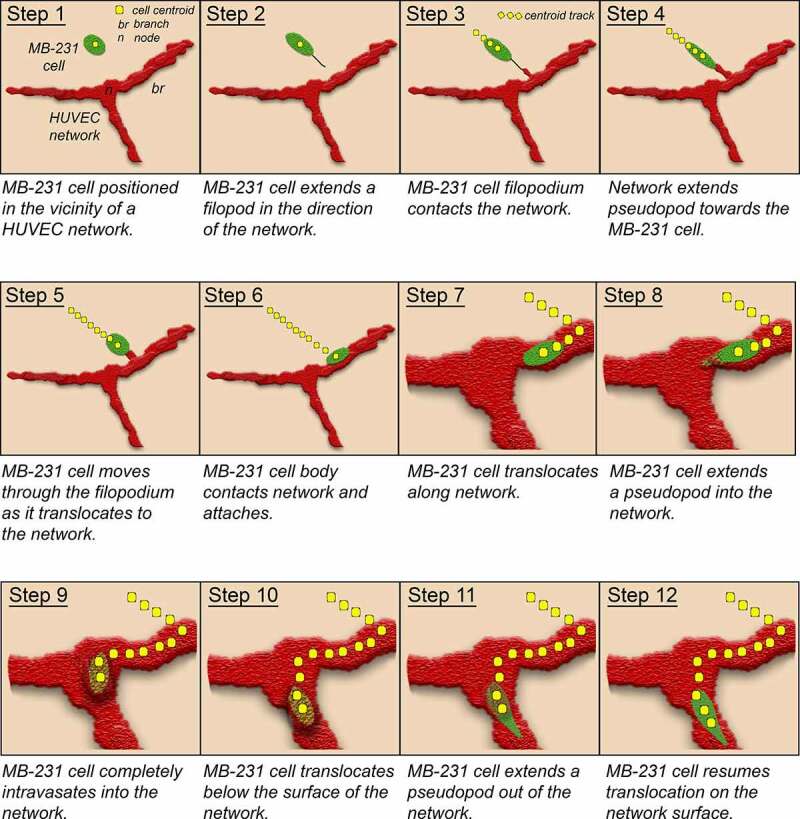
Model of the behaviors revealed here of tumorigenic MB-231 cells interacting with a HUVEC network. Green, MB-231 cells; red, HUVEC network; gold, MB-231 cell within the HUVEC network; yellow dots, centroid path of MB-231 cell; black line, filopod extended by MB-231 cell; red projection, cytoplasmic HUVEC projection connecting to the MB-231 filopod

Mammary tumor-derived MB-231 cells dispersed in a 3D Matrigel environment in the absence of HUVECs were motile, but nondirectional and did not coalesce into large aggregates during the initial 72 hours of analysis [26]. When in the vicinity of a HUVEC network, however, MB-231 cells moved in a directed fashion toward, and attached to, the reticulated multicellular HUVEC network within 8 hours of incubation. Directional movement toward the network proceeded within a distance of approximately 60 μm from the network, a distance of roughly three or more cell diameters. The Matrigel region in this zone became relatively devoid of MB-231 cells as they translocated toward and attached to the static HUVEC network. These behavioral characteristics differed markedly from those of MB-231 cells that are dispersed above a fibroblast monolayer [29]. In contrast to the stable network of HUVECs, the fibroblasts moved up from their network into the upper Matrigel layer and physically interacted with MB-231 cells, serving as scaffolds for MB-231 aggregation [29]. The mechanism of directional migration of MB-231 cells toward the HUVEC network in the *in vitro* model described here, could be due to chemotaxis, to a tactile mechanism, or to a combination of the two. In a positive chemotactic system, HUVECs would release a gradient of chemoattractant and the MB-231 cells would migrate up this gradient, in the direction of increasing chemoattractant concentration, by the biased extension of an anterior pseudopod or leading edge in the direction of the HUVEC network [71,72]. Vascular endothelial cells have been shown *in vitro* to release chemoattractants to which cancer cells respond by positive chemotaxis [73]. In an alternative tactile mechanism, filopodia which contact HUVECs would act as tactile sensors [74], expanding into pseudopods and directing cellular translocation toward the targeted HUVEC network [26,75]. Filopodia are actin-filled projections that can have diameters of 0.2 μm, extend up to three cell diameters in length [76] and expand into pseudopods [77]. Actin is organized in filopodia as bundles of unbranched filaments [78–80]. Our results suggest a tactile role for filopodia in directing MB-231 cell migration toward endothelial networks, but do not rule out an additional role for chemotaxis. Rather than function as a cable that is reeled into the HUVEC network, our observations suggest that the cell body expands into the attached filopod, by this means tracking its trajectory toward the HUVEC to which the filopod is attached. Our results also suggest that filopod contact elicits the endothelial target cell to extend a projection in the direction of the incoming MB-231 cell. Due to the low resolution of filopodia, we were unable to discriminate between filopod chemotaxis versus tactile filopod tracking. The behavior of a MB-231 cell moving toward, attaching to a HUVEC network, penetrating the network, translocating through the network and then exiting the network, is modeled in Figure 13.

### Behavior of MB-231 cells that attach to HUVEC networks

Both LSCM projections and J3D-DIAS reconstructions over time of live preparations revealed several behavioral characteristics of MB-231 cells after they have attached to HUVEC networks. First, once attached to the HUVEC network, MB-231 cells do not readily detach, suggesting that the adhesion forces to HUVECs are very strong. A number of endothelial adhesion molecules have been implicated in cancer cell-endothelium adhesion, including selectins, immunoglobulins, integrins and other binding proteins [59]. Second, although MB-231 cells attach tightly to the endothelial network, they are still highly motile, moving along the surface of network branches and nodes in a persistent fashion, stopping periodically. Small aggregates of MB-231 cells form on the HUVEC network, but motile cells frequently exit the aggregates, translocating along the network. Third, the HUVEC network appears static during attached MB-231 migration, suggesting that HUVECs function as a static scaffold for cancer cell migration. Fourth, MB-231 cells can penetrate HUVEC networks, translocate through them or under them, then reemerge and continue migration along the surface of the HUVEC networks (Figure 13). MB-231 cell penetration is mediated by an anterior pseudopod, or invadopod [81,82], that penetrates the multicellular endothelial network. Exiting the HUVEC network also appears to involve initially the appearance of the anterior pseudopod, followed by the cell body (Figure 13). These behaviors may in part mimic those of intravasation and extravasation during metastasis. Although the branches of the HUVEC network have been referred to as tubular [83], we saw no evidence in LSCM optical sections of tubular structure, and have assumed that the network, like capillary endothelial cells, maintains multicellular integrity through tight junctions [84,85]. It should be noted that the outside endothelial wall of capillaries is coated by basement membrane [86,87] which is similar in composition to Matrigel [88].

### The roles of CD44 and RHAMM

Studies show that CD44, a hyaluronic acid (HA) binding transmembrane glycoprotein, forms a complex with the nonintegral cell surface protein RHAMM [89–92] to promote tumor cell migration [93,94], and both may be upregulated in breast cancer [95,96]. Here, we tested the effects of mAbs against CD44 and RHAMM on MB-231 breast cancer cell behavior in our model. We observed that the relatively rapid motility along endothelial cells by MB-231 cancer cells was inhibited by treatment with antibodies against CD44 and RHAMM, and that the combinatorial effect on MB-231 cell velocity in Matrigel was greater than the effect of each Ab alone. Importantly, we noted that filopod formation was specifically inhibited in the presence of anti-RHAMM antibody, but not in the presence of anti-CD44 antibody, consistent with the reported stabilization of filopodia by RHAMM in squamous carcinoma cells [97]. Furthermore, we observed that over-expression of CD44 in MB-231 cells (MB-231 CD44°^e^) resulted in depletion of RHAMM from the membrane, loss of filopodia and concomitant inhibition of MB-231 cell behavior on HUVEC networks. Taken together, these data support the conclusion that a stoichiometric interaction between CD44 and RHAMM on the membrane is required to initiate invasive behaviors of cancer cells, and that an imbalance between the two may abrogate cancer cell metastatic potential, at least in part through the inhibition of filopod formation that arises, in turn, from the inactivation or mislocalization of RHAMM.

### Concluding remarks

In fixed sections of mouse mammary tumors formed by MB-231 cells, the MB-231 cells adhered to the outer surface of blood capillaries and exhibited the amorphic and elongate shapes of motile cells attached to HUVEC networks (Figure 12). Moreover, we found that fresh human breast cancer cells translocated to, adhered to, penetrated, translocated through and exited from HUVEC networks in our basic preparation. These results suggest that the behaviors described in our *in vitro* model may be representative of behaviors by breast cancer cells interacting with endothelial cells in the process of metastasis. This model provides not only a high resolution dynamic description of breast cancer-endothelial cell interactions, but also a vehicle for assessing the precise effect of antibodies against surface molecules involved in specific behaviors, such as directed movement facilitated by filopodia. However, the model we are developing is amenable to improvement, first by adding additional cell types found in natural tumors, such as macrophages and fibroblasts, and second by generating HUVEC tubules, such as those successfully formed in microfluidic chambers [15].

## Supplementary Material

Supplemental Material
