# Supplementary material for: Directed movement toward, translocation along, penetration into and exit from vascular networks by breast cancer cells in 3D
Source: Cell Adh Migr. 2021 Aug 2;15(1):224–48. doi: 10.1080/19336918.2021.1957527 (PMC8331046; doi:10.1080/19336918.2021.1957527)
Supplement: Supplemental Material [file KCAM_A_1957527_SM1394.zip › supplementary/Supplemental movie legend.docx]

Supplemental movie. Merged LSCM z-projections of MB-231-GFP breast cancer cells (green) interacting with a HUVEC network (red) at 10 minute intervals.
